# Preventing and Controlling Healthcare-Associated Infections: The First Principle of Every Antimicrobial Stewardship Program in Hospital Settings

**DOI:** 10.3390/antibiotics13090896

**Published:** 2024-09-20

**Authors:** Massimo Sartelli, Corrado P. Marini, John McNelis, Federico Coccolini, Caterina Rizzo, Francesco M. Labricciosa, Patrizio Petrone

**Affiliations:** 1 Department of Surgery, Macerata Hospital, 62100 Macerata, Italy; massimosartelli@infectionsinsurgery.org; 2 Jacobi Medical Center, New York Medical College, Bronx, NY 10461, USA; corradom@nychhc.org; 3 Jacobi Medical Center, Albert Einstein College of Medicine, Bronx, NY 10461, USA; john.mcnelis@nychhc.org; 4 General, Emergency and Trauma Surgery Unit, Pisa University Hospital, 56125 Pisa, Italy; federico.coccolini@unipi.it; 5 Department of Translational Research and New Technologies in Medicine and Surgery, University of Pisa, 56125 Pisa, Italy; caterina.rizzo@unipi.it; 6 Global Alliance for Infections in Surgery, 62100 Macerata, Italy; labricciosafrancesco@gmail.com; 7 NYU Langone Hospital—Long Island, NYU Grossman Long Island School of Medicine, Mineola, NY 11501, USA

**Keywords:** antimicrobial resistance, healthcare-associated infections, hospital-acquired infections, antimicrobial stewardship program, surveillance, infection control, prevention

## Abstract

Antimicrobial resistance (AMR) is one of the main public health global burdens of the 21st century, responsible for over a million deaths every year. Hospital programs aimed at improving antibiotic use, referred to as antimicrobial stewardship programs (ASPs), can both optimize the treatment of infections and minimize adverse antibiotics events including the development and spread of AMR. The challenge of AMR is closely linked to the development and spread of healthcare-associated infection (HAIs). In fact, the management of patients with HAIs frequently requires the administration of broader-spectrum antibiotic regimens due to the higher risk of acquiring multidrug-resistant organisms, which, in turn, promotes resistance. For this reason, even before using antibiotics correctly, it is necessary to prevent and control the spread of HAIs in our hospitals. In this narrative review, we present seven measures that healthcare workers, even if not directly involved in the tasks of infection prevention and control, must know, support, and embrace. We hope that this review may raise awareness among all healthcare professionals about the issues with the increasing rate of AMR and the ongoing efforts towards minimizing its rise.

## 1. Introduction

Healthcare-associated infections (HAIs), also known as nosocomial or hospital-acquired infections, which are preventable with the use of preventing strategies, are one of the potential risks to patient safety attributable to healthcare. They are associated with prolonged hospital stays, worse clinical outcomes, long-term disability, increased resistance of microorganisms to antimicrobials, massive costs, and unnecessary deaths [1]. HAIs continue to place a significant burden on the healthcare systems worldwide, causing high morbidity and mortality [2,3].

In recent years, point-prevalence studies from Europe were published [4,5]. A recent point-prevalence survey of HAIs and antimicrobial use in European acute care hospitals, performed in 2022–2023, estimated that 8.0% (95% confidence interval: 6.6–9.6%) of patients in acute care facilities experienced at least one HAI [4]. According to the European Centre for Disease Prevention and Control (ECDC), the burden of the six major types of HAIs in the European Union, expressed in disability-adjusted life years, was higher than the combined burden of all 32 other communicable diseases surveyed by the ECDC based on data from 2011 to 2012 [6]. Risk factors for the development of HAIs among hospitalized patients include patient-specific factors such as an immunocompromised condition and the need for chemotherapy and radiation therapy for cancer, as well as the overall intensity of treatment required, including the use of broad-spectrum antibiotics and a prolonged intensive care unit stay.

The HAI burden is closely related to the antimicrobial resistance (AMR). Notably, the management of patients with HAIs frequently requires the administration of broader-spectrum antibiotic regimens due to the higher risk of acquiring multidrug-resistant organisms (MDROs) [7,8], including methicillin-resistant *Staphylococcus aureus* (MRSA), vancomycin-resistant *Enterococcus faecium* (VRE), extended-spectrum beta-lactamase-producing *Enterobacterales* (ESBL) or carbapenemase-producing *Klebsiella pneumoniae*, and non-fermenting Gram-negative bacilli including *Pseudomonas aeruginosa*, *Acinetobacter baumannii,* and *Stenotrophomonas maltophilia* [1].

An in-depth analysis of the health impact of antibiotic resistance for 23 pathogens and 88 pathogen–antibiotic combinations in 204 countries using specific statistical models estimated that, in 2019, 4.95 million deaths were related to AMR, of which 1.27 million deaths were directly attributable to resistance [9]. Considering all ages, the highest mortality rate attributable to resistance was reported in Western Sub-Saharan Africa (27.3 deaths per 100,000 inhabitants) and the lowest in Australasia (6.5 deaths per 100,000 inhabitants). According to this study, six major pathogenic bacteria (*E. coli*, *S. aureus*, *K. pneumoniae*, *S. pneumoniae*, *P. aeruginosa*, and *A. baumannii*) caused 929,000 deaths attributable to antibiotic resistance and 3.57 million deaths related in some ways to antibiotic resistance. In particular, *S. aureus* methicillin resistance caused more than 100,000 deaths.

In recent years, the rate of fungal infections has been increasing, especially in at-risk populations, specifically, the immunosuppressed group, including HIV, cancer, and critically ill patients. Invasive fungal infections are characterized by high morbidity and mortality.

*Candida auris* has emerged worldwide as an MDRO [10,11,12]. The reasons are several: (1) its high transmissibility in hospital settings; (2) wide clinical manifestations associated with potentially high mortality; (3) environmental hardiness, including persistence for weeks on hospital surfaces due to biofilm formation; (4) difficulty in being identified by conventional microbiologic tests; and (5) high rate of antifungal resistance and treatment failure [8]. Given the growing concerns about proper infection management and the rising prevalence of HAIs [4,5], the adoption of an interdisciplinary and collaborative approach in acute and chronic healthcare facilities is imperative. Strategies for optimizing antimicrobial use should always entail a multifaceted approach, including measures to prevent and control HAIs before initiating antimicrobials. Within this context, antimicrobial stewardship programs (ASPs) are essential components of hospital infection control strategies, focusing on optimizing antimicrobial use to combat resistance and improve patient outcomes.

Recently, a call to action to optimize antibiotic use was published. The WARNING call to action proposed ten golden rules for using the right antibiotic for the right patient, at the right time, at the right dose, by the right route, and for the right duration [8]. The first golden rule for optimizing antibiotic use concerns strengthening infection prevention and control.

Preventing and controlling HAIs is a fundamental principle of ASPs in hospital settings. Specific and general measures aimed at reducing the risk of HAIs, combined with the strategic use of antimicrobials, are essential to improving patient safety and healthcare quality. This narrative review aims to present the main HAIs, and identify seven infection prevention and control strategies that every healthcare professional should know, support, and embrace in every hospital worldwide.

## 2. Methods

An international working group of physicians was enrolled by the Global Alliance for Infections in Surgery in order to define the most important measures to prevent and control HAIs in order to mitigate the spread of AMR. Seven preventive measures were defined by the working group.

Accurate searches were conducted using the PubMed^®^/MEDLINE (National Library of Medicine, Bethesda, MD, USA) databases to identify supporting evidence. Overall, 462 abstracts published in the English language between January 2008 and May 2024 were identified. Eighty-three articles were reviewed to prepare the first draft of the narrative review. The resulting document was shared with all the members of the working group, thoroughly reviewed, and finally approved.

## 3. Infection Prevention and Control Strategies

### 3.1. Prevention of Healthcare-Associated Infections

Surgical site infection (SSI) and four other types of infections, central line-associated bloodstream infection (CLABSI), catheter-associated urinary tract infection (CAUTI), ventilator-associated pneumonia/hospital-acquired pneumonia (VAP/HAP), and *Clostridioides difficile* infection (CDI), account for the majority of all the HAIs described in hospitals worldwide [1].

As most HAIs are preventable, such infections are considered an important indicator of the quality of care provided to patients. In 2018, Schreiber et al. undertook a meta-analysis of studies published between 2005 and 2016 to evaluate the impact of multimodal intervention approaches to reduce CAUTIs, CLABSIs, SSIs, VAP, and HAP in acute care hospitals or settings of care for chronic patients [13]. Of the 5226 articles identified, 144 were submitted to final analysis. The meta-analysis demonstrated that the implementation of a multimodal protocol could yield a 35% to 55% potential reduction in HAIs, independent of the country’s income level.

#### 3.1.1. Surgical Site Infections

SSIs are the result of several factors. All surgical sites may be contaminated by bacteria, but only a few surgical sites develop clinical SSIs. Colonization can occur when bacteria replicate and adhere to the surgical site. If the host immunity response is not sufficient to overcome the effects of colonization, SSIs occur. In most patients, the infection does not develop because the host defences can counteract the bacteria that colonize the surgical site; however, in some patients, host defences fail to protect them from developing SSIs.

Bacteria isolated from SSIs may differ depending on the contamination of the surgical procedure. In patients undergoing clean surgeries, in which the gastrointestinal, gynaecological, and respiratory tracts have not been entered, the bacteria of the resident skin population of the patient are the most frequent bacteria isolated. The density and composition of the skin’s indigenous microflora vary with anatomical site. A higher density of microorganisms resides in moist regions such as the axillae, groin, and between the toes. Of particular importance to the avoidance of superficial SSIs in the performance of clean procedures is prepping last the areas with high microbial counts, including the axilla, groin, perineal region, anus, and vagina, and discarding the sponge used for the prep. Additionally, the umbilicus, which is considered contaminated, should be prepped first, most often with the use of prep-solution-soaked cotton-tipped applicators. In clean/contaminated and contaminated procedures, in which the gastrointestinal, gynaecological, and respiratory tracts have been entered, the aerobic and anaerobic bacteria of the endogenous flora of the entered organ are the most frequently isolated bacteria.

The World Health Organization (WHO) [14,15,16] published guidelines for SSI prevention in 2016. The Centers for Disease Control and Prevention (CDC) updated the guidelines in 2017 [17]. In 2016, the American College of Surgeons (ACS) and the Surgical Infection Society (SIS) updated their joint guidelines for SSI prevention [18]. In 2019, the National Institute for Health and Care Excellence (NICE) published online its new guidelines for SSI management [19]. In 2023, a new set of joint guidelines for SSI prevention in acute care environments was jointly completed [20] by a multi-society collaboration promoted by the Society for Healthcare Epidemiology of America (SHEA). The 2016 WHO global guidelines for the prevention of SSI are based on systematic reviews of the scientific literature and provide evidence-based recommendations to support actions to improve clinical practice. These guidelines contain 29 recommendations. Thirteen recommendations refer to the prevention of SSIs in the preoperative period and sixteen to prevention during and after surgery. They range from preoperative simple precautions, such as the patients’ preoperative shower, screening and decolonization for MRSA, or the timing of peri-operative antibiotic prophylaxis, to intra-operative measures, such as the use of alcohol-based antiseptic agents for surgical site preparation, the decision not to remove hair, or, if absolutely necessary, the decision to remove it with a clipper, or the use of triclosan-coated sutures, up to postoperative measures such as interrupting antibiotic prophylaxis after surgery and correct management of the surgical wound.

#### 3.1.2. Catheter-Associated Urinary Tract Infections

Among all HAIs, urinary tract infections are the most common, and most of them are associated with the use of an indwelling urinary catheter. In recent years, CAUTIs have received less attention than other HAIs, probably because they are associated with lower morbidity and mortality, as well as having a lower impact in terms of costs. However, since they are very common, it is important to consider their large cumulative impact [21].

Of all patients, 10% to 25% require urinary catheters during hospitalization, and many of them develop a urinary infection [1]. For patients undergoing surgical interventions, the available guidelines [22,23] suggest avoiding the urinary catheter when possible or removing it as soon as possible. When a urinary catheter is placed, microorganisms can adhere to its surface, forming large colonies bonded together, and usually enclosed in a polymer matrix, but not as a biofilm. A biofilm is defined as a complex aggregation of microorganisms adhered to each other by an extracellular matrix composed of secreted products of organisms and/or components of the microorganisms themselves. The cells within the biofilm can be irreversibly bound to the surface. The biofilm may contain only one or more species of the microorganisms involved.

Prevention practices should include correct catheter placement techniques to minimize contamination and maintenance of a closed drainage system to avoid the contamination of the catheter. Multimodal interventions including the involvement and training of medical staff and nursing have proven to be more effective than a single intervention [24]. However, the two most important strategies for preventing CAUTI are (a) not using a urinary catheter, and (b) removing it promptly when it is no longer needed.

Systemic antibiotic prophylaxis should not be used routinely in patients with both short- and long-term catheterization, including patients undergoing surgical procedures. Asymptomatic bacteriuria does not require treatment with antibiotics [8]. Patients with a urinary catheter may have “positive” urine culture due to the inevitable biofilm formation on the catheter. Antibiotic administration is required in patients needing transurethral instrumentation.

#### 3.1.3. Hospital-Acquired Pneumonia and Ventilator-Associated Pneumonia

Nosocomial pneumonia is generally classified into hospital-acquired pneumonia (HAP) and ventilator-associated pneumonia (VAP). They are the second most frequent HAI, representing the first HAI in terms of morbidity and mortality.

The pathogenesis of HAP is multifactorial. Nosocomial pneumonia may be typically associated with colonization of the aero-digestive tract and the aspiration of contaminated secretions. Compromised host defences such as critical illnesses, comorbidities, drugs, and surgical procedures can contribute to the development of HAP.

Most cases of HAP are caused by aerobic Gram-negative bacteria, such as *P. aeruginosa* and *K. pneumoniae*. *S. aureus* is the most common among Gram-positive bacteria. The growing burden of antimicrobial resistance can make the treatment of pneumonia very difficult [25].

Many hospitalized patients with malnutrition and severe illness may be predisposed to a high rate of nosocomial pneumonia. Aspiration of oropharyngeal secretions can be associated with the development of nosocomial pneumonia.

Impaired function combined with reduced mucociliary clearance of the respiratory tract and its colonization can make aspiration a major contributor to nosocomial pneumonia. Aspiration may be facilitated by supine positioning.

The administration of stress ulcer prophylaxis, such as proton pump inhibitors (PPIs), commonly prescribed in critically ill patients, has been described to increase the risk of nosocomial pneumonia [26]. Finally, endotracheal and nasogastric tubes, essential to support critically ill patients, provide a source of colonization, leading to the migration of bacteria in the lower respiratory tract [27] and the development of ventilator-associated pneumonia (VAP).

It has been described that intubation and mechanical ventilation can increase the risk of VAP by 6 to 21 times. The duration of the mechanical ventilation and the need for reintubation are also described as important risk factors for the development of VAP. Other risk factors reported in the literature include body position, enteral feeding, ventilator support for more than seven days, and a Glasgow Coma Scale score of less than 9 [27,28,29]. The effects of oral hygiene care on the incidence of VAP in critically ill patients undergoing mechanical ventilation were described by a Cochrane systematic review in 2016 [30]. This review demonstrated that oral hygiene reduces the risk of VAP in critically ill patients. However, the review did not demonstrate any differences in mortality, duration of mechanical ventilation, or duration of intensive care unit stay.

#### 3.1.4. Central Line-Associated Bloodstream Infections

About half of HAIs occur in the intensive care unit and most of them are associated with intravascular devices [31]. The insertion of central venous catheters (CVCs) is very common in clinical practice. CVCs are inserted for the administration of fluids, blood products, drugs, and nutritional solutions and for hemodynamic monitoring of critically ill patients. They may be an important cause of bacteraemia in most hospitalized patients, and, therefore, CVCs should only be inserted if truly necessary.

The risk for CLABSIs is generally associated with patient, catheter, and procedure factors.

CVCs and arterial catheters are inserted in critically ill patients, including most often surgical patients. CVC-related complications include local complications at the site of insertion, infections, and thrombosis [28]. CLABSIs are responsible for high morbidity and mortality and additional costs.

The CDC have developed specific guidelines that are widely recognized as the document which best summarizes the current evidence for preventing CLABSIs [32]. A set of evidence-based guidelines was recently published for the management of intravascular catheters in intensive care units [33]. CLABSI prevention recommendations included the use of preferential catheterization of the subclavian vein, one-step skin disinfection using chlorhexidine in 2% alcohol solution, and the implementation of a quality improvement bundle protocol.

The use of catheters impregnated with antimicrobials using either antiseptic agents (chlorhexidine, silver sulfadiazine) or antibiotic agents (combination of minocycline and rifampicin) has been proposed to reduce the rate of CLABSIs in the literature. In 2016, a Cochrane meta-analysis was published comparing antimicrobial-impregnated CVCs to standard CVCs [34]. The meta-analysis failed to show a clear superiority of antimicrobial-impregnated CVCs over conventional catheters.

The occurrence of CLABSIs can be reduced by a set of simple measures, including the selection of the appropriate site, the use of closed infusion systems, the aseptic technique during insertion and management of the central venous catheter, using a lower number of lumens, and the early removal of central venous catheters.

#### 3.1.5. *Clostridioides difficile* Infections

In recent decades, the increase in the incidence of *Clostridioides difficile* in many countries has made it a global health problem. *C. difficile* is an anaerobic spore-forming Gram-positive bacillus which is part of the normal intestinal microbiota in healthy newborns. It may rarely be present in the intestines of healthy adults and spreads via the fecal–oral route. In hospitalized patients, it can be acquired through ingestion of spores or vegetative bacteria and spreads to patients through healthcare personnel, equipment, and environmental surfaces. CDI is a toxin-mediated infection; therefore, *C. difficile* strains not producing toxins are not pathogens [35].

Risk factors for developing CDI are divided into three general categories:

- Host-related factors (host immune status, related comorbidities);
- Exposure to *C. difficile* spores (hospitalizations, community sources, long-term hospitalizations);
- Factors that alter the normal microbiome of the colon (antibiotics, PPIs, surgery).

Antibiotic administration plays a crucial role in the pathogenesis of CDI through modification of the normal intestinal microbiome, providing a suitable environment for the proliferation of *C. difficile*. Although almost all antibiotics may be associated with CDI, clindamycin, third-generation cephalosporins, penicillins, and fluoroquinolones provide the highest risk for the development of CDI. The risk represented by the therapeutic use of proton pump inhibitors is unclear [35].

Rapid isolation of patients carrying *C. difficile* is crucial to control patient-to-patient transmission in hospital settings. It is particularly important to reduce environmental contamination since the spores can survive for months in the environment. Routine contact precautions in patients with CDI must be maintained until 48 h after the resolution of diarrhea. Patients with known or suspected CDI should be isolated in a room with a dedicated bathroom, if it is possible. If a single room is not available, patients with CDI can be cohorted in the same room with a dedicated bathroom or subjected to functional isolation [35].

Hand hygiene with soap and water and the use of contact precautions along with good environmental hospital hygiene should be respected by all healthcare personnel in contact with patients with known or suspected CDI [36]. Alcohol-based hand sanitizers are highly effective against non-spore-forming organisms, but are not effective against *C. difficile* spores.

### 3.2. Hand Hygiene

Hand hygiene is undoubtedly the cornerstone of effective infection prevention and control, representing the most critical measure to avoid the transmission of pathogenic microorganisms and prevent HAIs. A significant proportion of HAIs can be prevented by performing correct hand hygiene at appropriate times. Despite substantial evidence supporting the role of hand hygiene in reducing HAIs, numerous studies have shown that compliance with hand hygiene practices remains suboptimal [37]. Proper hand hygiene is a straightforward yet highly effective method for preventing and controlling the spread of infections. It is particularly crucial in preventing the spread of microorganisms, including MDROs, which pose significant treatment challenges.

Skin microorganisms can be categorized into transient and resident flora. Transient microorganisms reside on the outer layers of the skin and can be easily removed by hand hygiene. These transient microbes are often acquired by healthcare workers through direct contact with patients or contaminated surfaces and are most likely to cause HAIs.

In 2009, the WHO published comprehensive guidelines [38] providing healthcare professionals with specific recommendations to improve hand hygiene practices. More recently, the Society for Healthcare Epidemiology of America (SHEA), the Infectious Diseases Society of America (IDSA), and the Association for Professionals in Infection Control and Epidemiology (APIC) published joint practice recommendations for the prevention of HAIs through improved hand hygiene practices [39]. Hand hygiene can be performed using either soap and water or an alcohol-based hand rub. Hand washing with soap and water is recommended when hands are visibly soiled (including contamination with blood or other body fluids), after using the toilet, and when there is a potential exposure to spore-producing pathogens such as *C. difficile*. The water is not sufficient to remove hydrophobic substances such as fats and oils that may be present on dirty hands, necessitating the use of soaps. Non-antimicrobial soaps have minimal antimicrobial activity, while soaps containing antiseptic agents can inactivate or suppress skin microorganisms. Hands should be dried using disposable paper towels and the entire hand procedure should last 40–60 s. Hand rubbing with an alcohol-based solution has proven effective for hand hygiene, and is the preferred method when hands are not visibly soiled. Compared to soap and water, alcohol-based solution hand rubs are quicker, do not require infrastructure such as taps and clean water, and cause less skin irritation (most solutions contain emollients). The antimicrobial activity of alcohol results from its ability to denature proteins, with concentrations of 60–80%, demonstrating excellent germicidal activity against both Gram-positive and Gram-negative bacteria, including MDROs. However, the efficacy of hand hygiene products is influenced by several factors including the type of alcohol, its concentration, contact time, and the quantity of solution used. To ensure optimal hand hygiene, alcohol-based solution dispensers should be available at the patient’s bedside and in small portable bottles. Sanitizing hands with an alcohol-based solution typically requires 20–30 s.

### 3.3. Environmental Hospital Hygiene

Environmental hygiene is a pivotal measure in infection prevention and control in healthcare settings. Environmental contamination in hospital settings plays a key role in the transmission of HAIs [40], with estimates indicating that 20% of HAIs can originate from contaminated environmental surfaces [41].

Over the past few years, scientific evidence has demonstrated that contamination of surfaces in inpatient units significantly contributes to the transmission of microorganisms such as MRSA, VRE, *C. difficile*, and *Acinetobacter baumannii* [40].

A systematic review published in 2022 demonstrated the importance of improving hospital environmental hygiene to enhance patients’ safety. While most included studies were not of high quality, the review suggested that most environmental interventions were associated with lower rates of HAIs and/or patient colonization. Therefore, the implementation of cleaning and disinfection protocols is strongly recommended for high-touch surfaces to reduce the risk of outbreaks and the transmission of MDROs. Recent studies have shown that effective disinfection and cleaning are associated with a decrease in the environmental contamination of high-touch surfaces [42]. However, some evidence highlighted that the manual cleaning and disinfection of surfaces in hospitals are often suboptimal [43,44,45]. It is estimated that 5 to 30% of hospital surfaces remain contaminated despite the application of cleaning and disinfection protocols [42]. In addition, the use of contaminated cloths and/or solutions can promote the spread of microorganisms between different environments [46].

The ineffectiveness of cleaning can be attributed to the presence of the biofilm, where bacteria live protected for extended periods [47,48,49]. Hospital environmental hygiene is a complex process, influenced by several variables, including the product or intervention used, the technique and equipment employed, the type of surface, the level of environmental contamination, and the training of environmental hygiene personnel [50].

Disinfectants including alcohol, chlorine, aldehyde, amine, oxidatives (e.g., hydrogen peroxide, peracetic acid), and phenolic and quaternary ammonium compounds are commonly used in clinically practice. New products such as improved hydrogen peroxide liquid disinfectants, and peracetic acid–hydrogen peroxide combinations, are available and in development. Combined cleaning and disinfecting products also exist, and only products approved by regulatory authorities should be used.

An ideal disinfectant should be effective against bacteria, spores, and viruses, and, meanwhile, it should not have any impact on the environment and should be safe and easy to use. However, currently, an ideal product does not exist [51].

In recent years, there has been increased interest in the development of new disinfection technologies that, alone or in combination with traditional methods, can ensure containment. Examples include ultraviolet device systems for terminal room decontamination [52,53].

Given the lack of detailed protocols for the daily cleaning process, Dancer and Kramer proposed a simple four-step protocol (Look, Plan, Clean, Let Dry) for the cleaning process [54].

Effective cleaning and disinfection of the hospital environment require adequate staffing, equipment, training, and team communication [51].

### 3.4. Screening, Decolonization, Isolation, and Cohorting

Early detection of MDROs is an important component of any infection control program. Decolonization is a measure aimed at reducing or eliminating the bacterial burden to reduce the risk of infection. There is good evidence that active screening of preoperative patients for both methicillin-sensitive *S. aureus* and MRSA before cardiac and orthopaedic surgery, with the decolonization of carriers, results in reductions in postoperative infections caused by MRSA.

In an intensive care unit (ICU) setting, in 2013, a cluster-randomized, nonblinded trial demonstrated that daily bathing with a 2% chlorhexidine gluconate cloth resulted in a 23% decrease in VRE and MRSA acquisition and a 28% reduction in bloodstream infections [55].

To investigate the role of targeted versus universal decolonization in preventing ICU HAIs, a cluster-randomized trial was conducted in 43 hospitals, enrolling 74 ICUs and 74,256 patients. Hospitals were randomized to one of the following three strategies: MRSA screening and isolation; targeted decolonization including screening, isolation, and decolonization of MRSA carriers; and universal decolonization without screening and decolonization of all patients.

Universal decolonization resulted in higher effectiveness compared with targeted decolonization or screening and isolation in reducing MRSA rates, clinical isolates, and bloodstream infection [56]. Most decolonization interventions consist of a nasal product combined with aseptic bathing. Surveillance cultures for carbapenem-resistant *Enterobacterales* (CRE) are advocated in several reports and recommendations as part of an overall strategy to combat AMR [57].

Due to the limited antibiotic options and substantial mortality associated with infections caused by CRE, prevention is of the utmost importance. Underlying comorbidities, previous antimicrobial exposure, indwelling devices, and prior admission to healthcare facilities are major risk factors for CRE colonization. Active screening for CRE using rectal surveillance cultures is effective, when part of a comprehensive infection control initiative, in mitigating the spread of CRE in healthcare facilities.

The risk of CRE colonization should be individualized. It should be always assessed according to the local prevalence, the individual risk of acquisition, and any linkages with other healthcare providers.

Screening for carriage of CRE at admission should be suggested for the following patients [58]:

- Those who have been colonized or infected by CRE within the last 12 months;
- Those who have been hospitalized within the last 12 months;
- Those who have received antibiotics within the last 12 months;
- Those who had a known epidemiological link with a confirmed CRE carrier within the last 12 months;
- Those who are admitted to high-risk units, or have a major surgical abdominal intervention planned and/or are undergoing treatment with immunosuppressive treatment (e.g., patients with inflammatory bowel disease).

Isolation or cohorting [59] of colonized/infected patients is another crucial cornerstone of infection prevention and control. It aims to prevent the transmission of microorganisms from infected or colonized patients to other patients, hospital visitors, and healthcare workers, who may subsequently transmit microorganisms to other patients. The term ‘isolation’ generally implies placing patients in single-patient rooms (preferably with their own toilet facilities) when available. When isolation rooms are in short supply, patients should be cohorted, grouping together patients who are colonized/infected with the same organism to confine them to one area and prevent contact with other patients.

It has been demonstrated that, when a patient is colonized or infected with MDROs, the risk of acquiring these organisms for a patient newly admitted to the same room is increased [60].

A study enrolling 10,289 patients with HAIs occurring over seven years in four hospitals showed that the risk of acquiring an HAI was nearly 6-fold when a prior bed occupant was colonized or infected [61].

Isolating/cohorting a patient with highly resistant bacteria is beneficial in preventing patient-to-patient spread. These measures should be an integral part of any IPC program; however, they are often not applied consistently and rigorously because they are expensive, time consuming, and often uncomfortable for patients.

### 3.5. Adapting Evidence-Based Practices to the Local Context

Guidelines are an important tool for disseminating best practices. The incidence of HAIs can be reduced by adhering to guidelines [62]. The guidelines can reduce unjustified practices, improving the quality and safety of healthcare. They can be used to educate and train healthcare professionals.

While solid scientific evidence is available for the prevention of HAIs, compliance remains uniformly poor as a result of a widespread failure to accept the guidelines in daily clinical practice. Adapting evidence-based guidelines into a local context can improve acceptance and adherence to best practices. Importantly, guidelines alone are not sufficient to guarantee their use and the implementation of their principles. Every effort should be aimed at identifying adequate guidelines that can be applied locally without undue resistance on the part of practitioners.

Active user involvement in protocol creation can lead to wider acceptance in practice. Translating the recommendations into a local protocol or path that specifies responsibilities for particular actions in the hospital setting is one way to involve health workers directly.

In the context of a multimodal strategy, one of the most used methods to implement the prevention of HAIs is the bundle. The bundle is a contained set of interventions, evidence-based behaviors, and/or practices (from three to five) aimed at a specific type of patient and setting care which, applied jointly and appropriately, improves the quality and outcome of the processes with a greater effect than would be determined if each strategy were implemented separately.

Starting in 2001, the bundle concept was developed by the Institute for Healthcare Improvement (IHI) as a support to healthcare professionals to improve the care of patients undergoing specific high-risk treatments. Bundles used as part of multimodal strategies have reduced rates of all types of HAI [63,64,65,66,67,68,69,70,71,72,73,74,75]. Based on the guidelines, we suggest some measures that could be included in prevention bundles [76].

### 3.6. Surveillance

HAI surveillance includes the continuous and systematic collection, analysis, and interpretation of data on HAIs for planning, implementation, and evaluation of IPC practices, and is closely integrated with the timely dissemination of these data to relevant stakeholders.

It is widely acknowledged that surveillance systems allow both evaluation of the local burden of HAIs, measuring the effectiveness of strategies implemented to decrease HAI rates, and contribution to the early detection of HAIs including the identification of clusters and outbreaks [76].

Surveillance involves well-defined phases, including monitoring an event, collecting and analyzing data associated with the event, and feedbacking to the healthcare professionals involved [77]. It improves patient outcomes and also enables hospitals and healthcare professionals to evaluate the effectiveness of the implemented IPC strategies [78]. Feedback on results should be disseminated promptly to all levels of the organization.

Traditional surveillance methods, while effective, are resource intensive. The development of new technologies, such as artificial intelligence (AI), has the potential to support traditional surveillance by analyzing increasing numbers of health data and addressing patient needs [79]. Due to advances in technology, in recent years, automated surveillance protocols have led to improved performance, and increased accuracy, and overall improved patient safety [80].

A systematic review of artificial-intelligence-based tools to control HAI published in 2020 demonstrated that machine-learning-based models were associated with high performance standards for HAI surveillance [80]. Finally, there was a recent scoping review about new technologies applied to the surveillance, control, and prevention of HAIs in hospital settings [81]. Comparative analysis of new technologies and traditional methods showed that digital tools show promise in HAI surveillance, especially for SSIs; however, challenges persist in resource distribution and interdisciplinary integration in healthcare settings. This highlights the need for ongoing development and implementation strategies to maximize the benefits of these technologies [81].

### 3.7. Promoting Safety Culture

Safety culture can be defined as the result of individual and group beliefs, values, attitudes, perceptions, skills, and behavior patterns determining the organization’s commitment to patient safety [82]. There is evidence that driving large-scale improvement in infection and control practices and in decreasing HAI rates requires multifaceted strategies that address healthcare professionals’ knowledge, attitudes, and behaviors as well as organizational factors.

Healthcare professionals should be prepared to deal with complex systems to ensure the best interests of patients. At an individual level, each healthcare professional should have the necessary knowledge, skills, and abilities to implement effective infection prevention and control practices. However, the involvement of healthcare professionals in infection prevention and control and safety practices encounters complex organizational environments where resources are most of the time inadequate and the activity of health professionals is constantly overwhelmed by other demands. Furthermore, healthcare professionals often perceive infection prevention and control as marginal to their clinical role, and adherence to infection prevention and control practices is often inadequate among healthcare workers. Hospitals should have regular education and training programs on infection prevention and control for all healthcare professionals involved in the care of patients. However, integrating new interventions within one’s beliefs and perceptions and contextualizing them in the setting where they work is critical. Although it is very important to survey the application of prevention measures, in the context of a climate of collaboration, restrictive and punitive mandates should be avoided because they fail to achieve their stated objective. It should be very important to encourage an institutional culture of safety in which healthcare workers are persuaded, rather than forced, to comply with infection prevention measures. Hospitals with a strong safety culture can promote education, encourage communication and interdisciplinarity, and involve their healthcare professionals, promoting a positive, proactive, and collaborative climate [83]. Improvements in the prevention and control of HAIs improve the overall quality of patients’ care.

## 4. Conclusions

AMR is one of the main public health global burdens of the 21st century, resulting in a public health crisis which could threaten the practice of modern medicine. It is a natural phenomenon that occurs as microorganisms evolve. However, human practices have accelerated the pace at which microorganisms develop and spread AMR and the inappropriate use of antimicrobials in humans and animals has favored this phenomenon. The HAI burden is closely related to the AMR. Notably, the management of patients with HAIs frequently requires the administration of broader-spectrum antibiotic regimens due to the higher risk of acquiring MDROs.

Infection prevention and control practices in acute care hospitals are critical to help decrease the burden of HAIs and AMR. Preventing the occurrence and controlling the spread of HAIs should be considered the first principles of an appropriate antimicrobial stewardship program.

Hospital programs aimed at improving antimicrobial use, referred to as ASPs, can both optimize the treatment of infections and minimize adverse antimicrobial events, including the development and spread of AMR. Every hospital worldwide should invest existing resources to organize an effective antimicrobial stewardship team. However, the challenge of AMR is closely linked to HAIs. In fact, HAIs are often caused by MDROs.

For this reason, even before using antimicrobials correctly, it is necessary to prevent and control the spread of HAIs in our hospitals. In this narrative review, we have presented seven measures that all healthcare workers, even if not directly involved in the task of infection prevention and control, must know, support, and embrace. We hope that this review can contribute to raising awareness among healthcare professionals about all issues associated with HAIs and the need to embrace personal involvement in the strategies that can help reduce their occurrence.

The fewer microorganisms there are in our hospitals, the fewer antimicrobials we will use, the fewer HAIs will develop in our hospitals, and the fewer broad-spectrum antibiotics will be required to treat MDROs.

## References

[1] Haque M., Sartelli M., McKimm J., Abu Bakar M. (2018). Health care-associated infections—An overview. Infect. Drug Resist..

[2] Su L.H., Chen I.L., Tang Y.F., Lee J.S., Liu J.W. (2020). Increased financial burdens and lengths of stay in patients with healthcare-associated infections due to multidrug-resistant bacteria in intensive care units: A propensity-matched case-control study. PLoS ONE.

[3] Valiquette L., Chakra C.N., Laupland K.B. (2014). Financial impact of health care-associated infections: When money talks. Can. J. Infect. Dis. Med. Microbiol..

[4] ECDC Surveillance Report. Point Prevalence Survey of Healthcare Associated Infections and Antimicrobial Use in European Acute Care Hospitals. 2022–2023. https://www.ecdc.europa.eu/sites/default/files/documents/healthcare-associated-point-prevalence-survey-acute-care-hospitals-2022-2023.pdf.

[5] Mitchell R., Taylor G., Rudnick W., Alexandre S., Bush K., Forrester L., Frenette C., Granfield B., Gravel-Tropper D., Happe J. (2019). Canadian Nosocomial Infection Surveillance Program. Trends in health care-associated infections in acute care hospitals in Canada: An analysis of repeated point-prevalence surveys. Can. Med. Assoc. J..

[6] Cassini A., Plachouras D., Eckmanns T., Abu Sin M., Blank H.P., Ducomble T., Haller S., Harder T., Klingeberg A., Sixtensson M. (2016). Burden of six healthcare-associated infections on European population health: Estimating incidence-based disability-adjusted life years through a population prevalence-based modelling study. PLoS Med..

[7] Canadian Nosocomial Infection Surveillance Program (2023). Healthcare-associated infections and antimicrobial resistance in Canadian acute care hospitals, 2017–2021. Can. Commun. Dis. Rep..

[8] Worldwide Antimicrobial Resistance National/International Network Group (WARNING) Collaborators (2023). Ten golden rules for optimal antibiotic use in hospital settings: The WARNING call to action. World J. Emerg. Surg..

[9] Antimicrobial Resistance Collaborators (2022). Global burden of bacterial antimicrobial resistance in 2019: A systematic analysis. Lancet.

[10] Dahiya S., Chhillar A.K., Sharma N., Choudhary P., Punia A., Balhara M., Kaushik K., Parmar V.S. (2020). Candida auris and nosocomial infection. Curr. Drug Targets..

[11] Dubey A.K., Singla R.K. (2019). Perspectives on anti-Candida drug development. Curr. Top. Med. Chem..

[12] Lyman M., Forsberg K., Sexton D.J., Chow N.A., Lockhart S.R., Jackson B.R., Chiller T. (2023). Worsening spread of Candida auris in the United States, 2019 to 2021. Ann Intern Med..

[13] Schreiber P.W., Sax H., Wolfensberger A., Clack L., Kuster S.P., Swissnoso. (2018). The preventable proportion of healthcare- associated infections 2005–2016: Systematic review and meta-analysis. Infect. Control Hosp. Epidemiol..

[14] Allegranzi B., Zayed B., Bischoff P., Kubilay N.Z., de Jonge S., de Vries F., Gomes S.M., Gans S., Wallert E.D., Wu X. (2016). New WHO recommendations on intraoperative and postoperative measures for surgical site infection prevention: An evidence-based global perspective. Lancet Infect. Dis..

[15] Allegranzi B., Bischoff P., de Jonge S., Kubilay N.Z., Zayed B., Gomes S.M., Abbas M., Atema J.J., Gans S., van Rijen M. (2016). New WHO recommendations on preoperative measures for surgical site infection prevention: An evidence-based global perspective. Lancet Infect. Dis..

[16] WHO (2016). Global Guidelines for the Prevention of Surgical Site Infection.

[17] Berríos-Torres S.I., Umscheid C.A., Bratzler D.W., Leas B., Stone E.C., Kelz R.R., Reinke C.E., Morgan S., Solomkin J.S., Mazuski J.E. (2017). Centers for disease control and prevention guideline for the prevention of surgical site infection, 2017. JAMA Surg..

[18] Ban K.A., Minei J.P., Laronga C., Harbrecht B.G., Jensen E.H., Fry D.E., Itani K.M., Dellinger E.P., Ko C.Y., Duane T.M. (2017). American College of Surgeons and Surgical Infection Society: Surgical site infection guidelines, 2016 update. J. Am. Coll. Surg..

[19] National Institute for Health and Care Excellence Surgical Site Infections: Prevention and Treatment. NICE Guideline [NG125]. https://www.nice.org.uk/guidance/ng125.

[20] Calderwood M.S., Anderson D.J., Bratzler D.W., Dellinger E.P., Garcia-Houchins S., Maragakis L.L., Nyquist A.C., Perkins K.M., Preas M.A., Saiman L. (2023). Strategies to prevent surgical site infections in acute-care hospitals: 2022 Update. Infect. Control Hosp. Epidemiol..

[21] Gad M.H., AbdelAziz H.H. (2021). Catheter-associated urinary tract infections in the adult patient group: A qualitative systematic review on the adopted preventative and interventional protocols from the literature. Cureus.

[22] Gould C.V., Umscheid C.A., Agarwal R.K., Kuntz G., Pegues D.A., Healthcare Infection Control Practices Advisry Committee (2010). Healthcare Infection Control Practices Advisory Committee. Guideline for prevention of catheter-associated urinary tract infections 2009. Infect. Control Hosp. Epidemiol..

[23] Hooton T.M., Bradley S.F., Cardenas D.D., Colgan R., Geerlings S.E., Rice J.C., Saint S., Schaeffer A.J., Tambayh P.A., Tenke P. (2010). Diagnosis, prevention, and treatment of catheter-associated urinary tract infection in adults: 2009 international clinical practice guidelines from the infectious diseases society of America. Clin. Infect. Dis..

[24] Parker V., Giles M., Graham L., Suthers B., Watts W., O’Brien T., Searles A. (2017). Avoiding inappropriate urinary catheter use and catheter- associated urinary tract infection (CAUTI): A pre-post control intervention study. BMC Health Serv. Res..

[25] Rotstein C., Evans G., Born A., Grossman R., Light R.B., Magder S., McTaggart B., Weiss K., Zhanel G.G. (2008). Clinical practice guidelines for hospital-acquired pneumonia and ventilator-associated pneumonia in adults. Can. J. Infect. Dis. Med. Microbiol..

[26] Abad C.L., Formalejo C.P., Mantaring D.M. (2021). Assessment of knowledge and implementation practices of the ventilator acquired pneumonia (VAP) bundle in the intensive care unit of a private hospital. Antimicrob. Resist. Infect. Control.

[27] Rello J., Afonso E., Lisboa T., Balsera B., Rovira A., Valles J., Diaz E., FADO Project Investigator (2013). A care bundle approach for prevention of ventilator-associated pneumonia. Clin. Microbiol. Infect..

[28] Klompas M., Branson R., Cawcutt K., Eichenwald E.C., Greene L.R., Lee G., Maragakis L.L., Powell K., Priebe G.P., Speck K. (2022). Strategies to prevent ventilator-associated pneumonia in acute care hospitals: 2022 Update. Infect. Control Hosp. Epidemiol..

[29] Torres A., Niederman M.S., Chastre J., Ewig S., Fernandez-Vandellos P., Hanberger H., Kollef M., Bassi G.L., Luna C.M., Martin-Loeches I. (2017). International ERS/ESICM/ESCMID/ALAT guidelines for the management of hospital-acquired pneumonia and ventilator-associated pneumonia. Eur. Respir. J..

[30] Hua F., Xie H., Worthington H.V., Furness S., Zhang Q., Li C. (2016). Oral hygiene care for critically ill patients to prevent ventilator-associated pneumonia. Cochrane Database Syst. Rev..

[31] Patil H.V., Patil V.C., Ramteerthkar M.N., Kulkami R.D. (2011). Central venous catheter-related bloodstream infections in the intensive care unit. Indian. J. Crit. Care Med..

[32] Gahlot R., Nigam C., Kumar V., Yadav G., Anupurba S. (2014). Catheter-related bloodstream infections. Int. J. Crit. Illn. Inj. Sci..

[33] O’Grady N.P., Alexander M., Burns L.A., Dellinger E.P., Garland J., Heard S.O., Lipsett P.A., Masur H., Mermel L.A., Perason M.L. (2011). Guidelines for the prevention of intravascular catheter-related infections. Clin. Infect. Dis..

[34] Timsit J.F., Baleine J., Bernard L., Calvino-Gunther S., Darmon M., Dellamonica J., Desruennes E., Leone M., Lepape A., Leroy O. (2020). Expert consensus-based clinical practice guidelines management of intravascular catheters in the intensive care unit. Ann. Intensive Care..

[35] Sartelli M., Di Bella S., McFarland L.V., Khanna S., Furuya-Kanamori L., Abuzeid N., Abu-Zidan F., Ansaloni L., Augustin G., Bala M. (2019). 2019 update of the WSES guidelines for management of Clostridioides (Clostridium) difficile infection in surgical patients. World J Emerg Surg..

[36] Oughton M.T., Loo V.G., Dendukuri N., Fenn S., Libman M.D. (2009). Hand hygiene with soap and water is superior to alcohol rub and antiseptic wipes for removal of Clostridium difficile. Infect. Control Hosp. Epidemiol..

[37] Toney-Butler T.J., Gasner A., Carver N. (2024). Hand Hygiene. StatPearls.

[38] WHO (2009). WHO Guidelines on Hand Hygiene in Health Care.

[39] Glowicz J.B., Landon E., Sickbert-Bennett E.E., Aiello A.E., deKay K., Hoffmann K.K., Maragakis L., Olmsted R.N., Polgree P.M., Trexler P.A. (2023). SHEA/IDSA/APIC practice recommendation: Strategies to prevent healthcare-associated infections through hand hygiene: 2022 Update. Infect. Control Hosp. Epidemiol..

[40] Otter J.A., Yezli S., Salkeld J.A., French G.L. (2013). Evidence that contaminated surfaces contribute to the transmission of hospital pathogens and an overview of strategies to address contaminated surfaces in hospital settings. Am. J. Infect. Control.

[41] Weber D.J., Rutala W.A., Miller M.B., Huslage K., Sickbert-Bennett E. (2010). Role of hospital surfaces in the transmission of emerging health care-associated pathogens: Norovirus, Clostridium difficile, and Acinetobacter species. Am. J. Infect. Control.

[42] Carling P.C., Bartley J.M. (2010). Evaluating hygienic cleaning in health care settings: What you do not know can harm your patients. Am. J. Infect. Control.

[43] Dancer S.J. (2008). Importance of the environment in meticillin-resistant *Staphylococcus aureus* acquisition: The case for hospital cleaning. Lancet Infect. Dis..

[44] Carling P.C., Parry M.M., Rupp M.E., Po J.L., Dick B., Von Beheren S. (2008). Improving cleaning of the environment surrounding patients in 36 acute care hospitals. Infect. Control Hosp. Epidemiol..

[45] Casini B., Tuvo B., Cristina M.L., Spagnolo A.M., Totaro M., Baggiani A., Privitera G.P. (2019). Evaluation of an Ultraviolet C (UVC) Light-Emitting Device for Disinfection of High Touch Surfaces in Hospital Critical Areas. Int. J. Environ. Res. Public Health.

[46] Casini B., Tuvo B., Scarpaci M., Totaro M., Badalucco F., Briani S., Luchini G., Costa A.L., Baggiani A. (2023). Implementation of an Environmental Cleaning Protocol in Hospital Critical Areas Using a UV-C Disinfection Robot. Int. J. Environ. Res. Public Health.

[47] Vickery K., Deva A., Jacombs A., Allan J., Valente P., Gosbell I.B. (2012). Presence of biofilm containing viable multiresistant organisms despite terminal cleaning on clinical surfaces in an intensive care unit. J. Hosp. Infect..

[48] Hu H., Johani K., Gosbell I.B., Jacombs A.S.W., Almatroudi A., Whiteley G.S., Deva A.K., Jensen S., Vickery K. (2015). Intensive care unit environmental surfaces are contaminated by multidrug-resistant bacteria in biofilms: Combined results of conventional culture, pyrosequencing, scanning electron microscopy, and confocal laser microscopy. J. Hosp. Infect..

[49] Otter J.A., Vickery K., Walker J.T., de Lancey Pulcini E., Stoodley P., Goldenberg S.D., Salkeld J.A.G., Chewins J., Yezli S., Edgeworth J.D. (2015). Surface-attached cells, biofilms and biocide susceptibility: Implications for hospital cleaning and disinfection. J. Hosp. Infect..

[50] Peters A., Otter J., Moldovan A., Parneix P., Voss A., Pittet D. (2018). Keeping hospitals clean and safe without breaking the bank; summary of the Healthcare Cleaning Forum 2018. Antimicrob. Resist. Infect. Control.

[51] Assadian O., Harbarth S., Vos M., Knobloch J.K., Asensio A., Widmer A.F. (2021). Practical recommendations for routine cleaning and disinfection procedures in healthcare institutions: A narrative review. J. Hosp. Infect..

[52] Weber D.J., Rutala W.A., Anderson D.J., Chen L.F., Sickbert-Bennett E.E., Boyce J.M. (2016). Effectiveness of ultraviolet devices and hydrogen peroxide systems for terminal room decontamination: Focus on clinical trials. Am. J. Infect. Control.

[53] Deshpande A., Mana T.S.C., Cadnum J.L., Jencson A.C., Sitzlar B., Fertelli D., Hurless K., Kundrapu S., Sunkesula V.C.K., Donskey C.J. (2014). Evaluation of a sporicidal peracetic acid/hydrogen peroxide-based daily disinfectant cleaner. Infect. Control Hosp. Epidemiol..

[54] Dancer S.J., Kramer A. (2019). Four steps to clean hospitals: LOOK, PLAN, CLEAN and DRY. J. Hosp. Infect..

[55] Climo M.W., Yokee D.S., Warren D.K., Perl T.M., Bolon M., Herwaldt L.A., Weinstein R.A., Sepkowitz K.A., Jernigan J.A., Sanogo K. (2013). Effect of daily chlorhexidine bathing on hospital-acquired infection. N. Engl. J. Med..

[56] Huang S.S., Septimus E., Kleinman K., Moody J., Hickok J., Avery T.R., Lankiewicz J., Gombosev A., Terpstra L., Hartford F. (2013). CDC Prevention Epicenters Program; AHRQ DECIDE Network and Healthcare-Associated Infections Program. Targeted versus universal decolonization to prevent ICU infection. N. Engl. J. Med..

[57] Richter S.S., Marchaim D. (2017). Screening for carbapenem-resistant Enterobacteriaceae: Who, When, and How?. Virulence.

[58] Sartelli M., Tascini C., Coccolini F., Dellai F., Ansaloni L., Antonelli M., Bartoletti M., Bassetti M., Boncagni F., Carlini M. (2024). Management of intra-abdominal infections: Recommendations by the Italian council for the optimization of antimicrobial use. World J. Emerg. Surg..

[59] WHO (2017). Guidelines for the Prevention and Control of Carbapenem-Resistant Enterobacteriaceae, Acinetobacter baumannii and Pseudomonas aeruginosa in Health Care Facilities.

[60] Chen L.F., Knelson L.P., Gergen M.F., Better O.M., Nicholson B.P., Woods C.W., Rutala W.A., Weber D.J., Sexton D.J., Anderson D.J. (2018). A prospective study of transmission of multidrug-resistant organisms (MDROs) between environmental sites and hospitalized patients—The TransFER study. Infect. Control Hosp. Epidemiol..

[61] Cohen B., Liu J., Cohen A.R., Larson E. (2018). Association between healthcare-associated infection and exposure to hospital roommates and previous bed occupants with the same organism. Infect. Control Hosp. Epidemiol..

[62] Flodgren G., Conterno L.O., Mayhew A., Omar O., Pereira C.R., Shepperd S. (2013). Interventions to improve professional adherence to guidelines for prevention of device-related infections. Cochrane Database Syst. Rev..

[63] Thandar M.M., Matsuoka S., Rahman, Ota E., Baba T. (2021). Infection control teams for reducing healthcare- associated infections in hospitals and other healthcare settings: A protocol for systematic review. BMJ Open.

[64] Dumyati G., Concannon C., van Wijngaarden E., Love T.M.T., Graman P., Pettis A.M., Greene L., El-Daher N., Farnsworth D., Quinlan G. (2014). Sustained reduction of central line- associated bloodstream infections outside the intensive care unit with a multimodal intervention focusing on central line maintenance. Am. J. Infect. Control.

[65] Jeong I.S., Park S.M., Lee J.M., Song J.Y., Lee S.J. (2013). Effect of central line bundle on central line-associated bloodstream infections in intensive care units. Am. J. Infect. Control.

[66] Klintworth G., Stafford J., O’Connor M., Leong T., Hamley L., Watson K., Kennon J., Bass P., Cheng A.C., Worth L.J. (2014). Beyond the intensive care unit bundle: Implementation of a successful hospital-wide initiative to reduce central line-associated bloodstream infections. Am. J. Infect. Control.

[67] Hakko E., Guvenc S., Karaman I., Cakmak A., Erdem T., Cakmakci M. (2015). Long-term sustainability of zero central-line associated bloodstream infections is possible with high compliance with care bundle elements. East. Mediterr. Health J..

[68] Jones C.M., Stewart C., Roszell S.S. (2015). Beyond best practice: Implementing a unit- based CLABSI project. J. Nurs. Care Qual..

[69] Al-Thaqafy M.S., El-Saed A., Arabi Y.M., Balkhy H.H. (2014). Association of compliance of ventilator bundle with incidence of ventilator-associated pneumonia and ventilator utilization among critical patients over 4 years. Ann. Thorac. Med..

[70] El Azab S.R., El Sayed A.E., Abdelkarim M., Al Mutairi K.B., Al Saqabi A., El Demerdash S. (2013). Combination of ventilator care bundle and regular oral care with chlorhexidine was associated with reduction in ventilator associated pneumonia. Egypt. J. Anaesthes..

[71] Micik S., Besic N., Johnson N., Han M., Hamlyn S., Ball H. (2013). Reducing risk for ventilator associated pneumonia through nursing sensitive interventions. Intensive Crit. Care Nurs..

[72] Tanner J., Padley W., Assadian O., Leaper D., Kiernan M., Edmiston C. (2015). Do surgical care bundles reduce the risk of surgical site infections in patients undergoing colorectal surgery? A systematic review and cohort meta-analysis of 8515 patients. Surgery.

[73] Ramsay G., Watson A. (2022). Reducing surgical site infection rates in colorectal surgery—A quality improvement approach to implementing a comprehensive bundle. Color. Dis..

[74] Izzo I., Lania D., Bella D., Marioni C.F., Coccaglio R., Colombini P. (2015). Catheter associated urinary tract infection (CA-UTI) incidence in an internal medicine ward of a northern Italian Hospital. Infez. Med..

[75] Saint S., Greene M.T., Krein S.L., Rogers M.A.M., Ratz D., Fowler K.E., Edson B.S., Watson S.R., Meyer-Lucas B., Masuga M. (2016). A Program to Prevent Catheter-Associated Urinary Tract Infection in Acute Care. N. Eng. J. Med..

[76] Sartelli M., Bartoli S., Borghi F., Busani S., Carsetti A., Catena F., Cillara N., Coccolini F., Cortegiani A., Cortese F. (2023). Implementation Strategies for Preventing Healthcare-Associated Infections across the Surgical Pathway: An Italian Multisociety Document. Antibiotics.

[77] Gastmeier P., Schwab F., Sohr D., Behnke M., Geffers C. (2009). Reproducibility of the surveillance effect to decrease nosocomial infection rates. Infect. Control Hosp. Epidemiol..

[78] Condon R.E., Schulte W.J., Malangoni M.A., Anderson-Teschendorf M.J. (1983). Effectiveness of a surgical wound surveillance program. Arch. Surg..

[79] Arzilli G., De Vita E., Pasquale M., Carloni L.M., Pellegrini M., Di Giacomo M., Esposito E., Porretta A.D., Rizzo C. (2024). Innovative Techniques for Infection Control and Surveillance in Hospital Settings and Long-Term Care Facilities: A Scoping Review. Antibiotics.

[80] Woeltje K.F., Lin M.Y., Klompas M., Wright M.O., Zuccotti G., Trick W.E. (2014). Data requirements for electronic surveillance of healthcare-associated infections. Infect. Control Hosp. Epidemiol..

[81] Scardoni A., Balzarini F., Signorelli C., Cabitza F., Odone A. (2020). Artificial intelligence-based tools to control healthcare associated infections: A systematic review of the literature. J. Infect. Public Health.

[82] Braun B.I., Chitavi S.O., Suzuki H., Soyemi C.A., Puig-Asensio M. (2020). Culture of Safety: Impact on Improvement in Infection Prevention Process and Outcomes. Curr. Infect. Dis. Rep..

[83] van Buijtene A., Foster D. (2019). Does a hospital culture influence adherence to infection prevention and control and rates of healthcare associated infection? A literature review. J. Infect. Prev..

